# Crystal structure of *catena*-poly[[[(2-eth­oxy­pyrazine-κ*N*)copper(I)]-di-μ_2_-cyanido] [copper(I)-μ_2_-cyanido]]

**DOI:** 10.1107/S205698901901452X

**Published:** 2019-10-31

**Authors:** Sofiia V. Partsevska, Valerii Y. Sirenko, Kateryna V. Terebilenko, Sergey O. Malinkin, Il’ya A. Gural’skiy

**Affiliations:** a Department of Chemistry, Taras Shevchenko National University of Kyiv, Volodymyrska St. 64, Kyiv 01601, Ukraine

**Keywords:** crystal structure, eth­oxy­pyrazine, cyanides, copper(I), metal–organic framework

## Abstract

In the asymmetric unit of the title coordination compound, {[Cu(CN)(C_4_H_3_OC_2_H_5_N_2_)][Cu(CN)]}_*n*_, there are two Cu atoms with different coordination environments. One Cu^I^ ion is coordinated in a triangular coordination geometry by the N atom of the 2-eth­oxy­pyrazine mol­ecule and by two bridging cyanide ligands, equally disordered over two sites exchanging C and N atoms, thus forming polymeric chains parallel to the *c* axis. The other Cu atom is connected to two bridging cyanide groups disordered over two sites with an occupancy of 0.5 for each C and N atom, and forming an almost linear polymeric chain parallel to the *b* axis. In the crystal, the two types of chain, which are orthogonal to each other, are connected by cuprophilic Cu⋯Cu inter­actions [2.7958 (13) Å], forming two-dimensional metal–organic coordination layers parallel to the *bc* plane. The coordination framework is further stabilized by weak long-range (electrostatic type) C—H⋯π inter­actions between cyano groups and 2-eth­oxy­pyrazine rings.

## Abstract 2

The title compound, {[Cu(EtOpz)(CN)_2_][CuCN]}_*n*_, where EtOpz is 2-eth­oxy­pyrazine, is a two-dimensional polymeric copper complex with different coordination environments of the two Cu^I^ ions. One Cu atom is coordinated to the 2-eth­oxy­pyrazine mol­ecule and two bridging cyanide ligands, equally disordered over two sites. The second Cu atom is coordinated by two disordered over two sites bridging cyanide groups. Two copper–cyanide chains are connected through Cu⋯Cu contact.

## Chemical context   

The design and synthesis of coordination polymers has received much attention in the field of inorganic chemistry due to their structural features, as well as their potential applications in catalysis, adsorption, luminescence and as chemical sensors (Li *et al.*, 2012 ▸; Czaja *et al.*, 2009 ▸; Etaiw *et al.*, 2016 ▸; Ley *et al.*, 2010 ▸). Complexes with the cyano group, which is one of the important bridging and assembling ligands acting as a monodentate, bidentate or tridentate ligand, are the subject of much inter­est (Ley *et al.*, 2010 ▸). Different types of metal cyanides with building blocks from linear *M*(CN)_2_ (Okabayashi *et al.*, 2009 ▸), trigonal *M*(CN)_3_ (Su *et al.*, 2011 ▸), tetra­hedral *M*(CN)_4_ (Jószai *et al.*, 2005 ▸) to high connected *M*(CN)_7_ (Qian *et al.*, 2013 ▸) and *M*(CN)_8_ (Chorazy *et al.*, 2013 ▸) units have been reported with various metal ions. Among the large number of various metal cyanides, copper(I) cyanide complexes are very important in organic, organometallic and supra­molecular chemistry because of both the copper centre, which possesses several coordination modes (two-, three-, four-, five- or six-coordinate) and can form diverse geometries, and the versatile cyanide ligand (Pike, 2012 ▸). In general, the crystallochemistry of Cu^I^CN systems is highly complex and provides several recurrent structural motifs: (i) linear chains similar to those of pure CuCN with possible disorder in the cyanide groups; (ii) six CN ligands connected by copper dimers with stoichiometry Cu_2_(1,1,2-μ_3_-CN)_2_(CN)_4_ and Cu⋯Cu distances typical of cuprophilic inter­actions; (iii) (CuCN)_*x*_ rings with square, penta­gonal or hexa­gonal geometry (Grifasi *et al.*, 2016 ▸; Pike, 2012 ▸). Mixed-valence Cu^I^/Cu^II^ coor­dination complexes with cyanide and amine ligands having different supra­molecular architectures and their luminescence properties have also been reported (Grifasi *et al.*, 2016 ▸). To improve the design of copper cyanide coordination polymers, as well as to investigate its influence on the resulting luminescence and other properties, different types of co-ligands were used, in particular, *N*-donor bridging or chelating ligands, such as 1,10-phenanthroline, 4,4′-bi­pyridine (Su *et al.*, 2011 ▸), pyridines with methyl, ethyl, meth­oxy and other substituents (Dembo *et al.*, 2010 ▸), and pyrazine (Qin *et al.*, 2012 ▸; Chesnut *et al.*, 2001 ▸) and its derivatives (Chesnut *et al.*, 2001 ▸). Here we describe the crystal structure of a new [CuCN]-based metal–organic coordination framework of the general formula {[Cu(CN)_2_(EtOpz)][CuCN]}_*n*_ (where EtOpz is 2-eth­oxy­pyrazine).
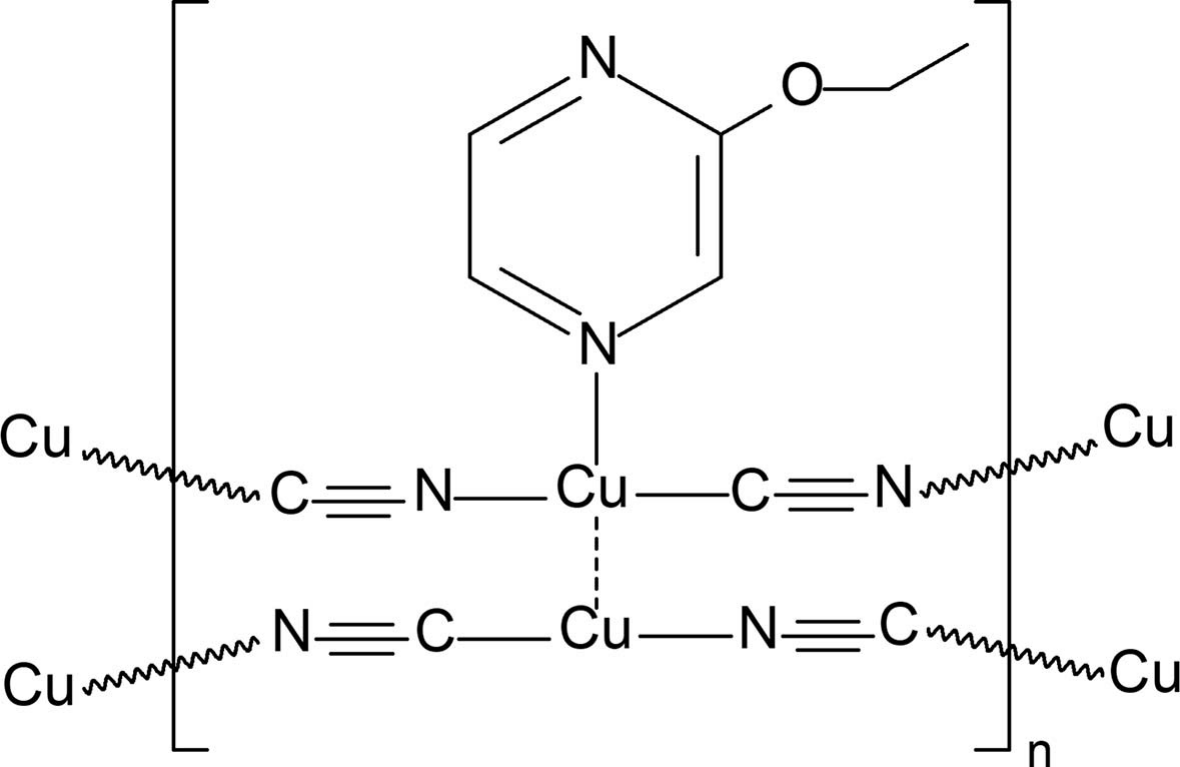


## Structural commentary   

**Figure 1:**
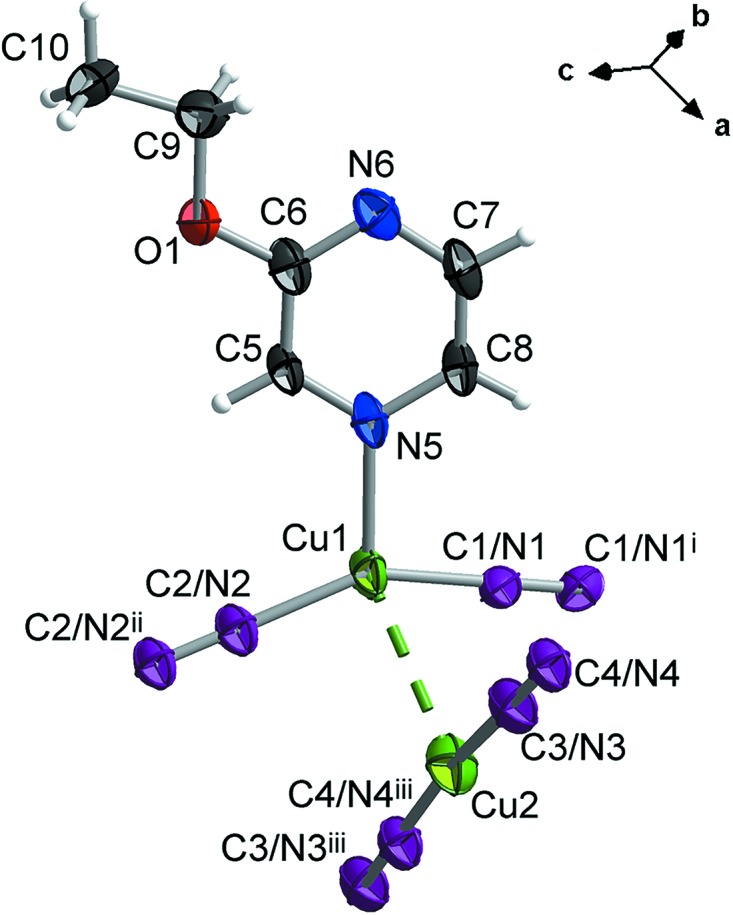
A fragment of the crystal structure of the title compound, with displacement ellipsoids drawn at the 65% probability level [symmetry codes: (i) −*x* + 1, *y*, −*z* + 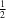; (ii) −*x* + 1, −*y*, −*z* + 1; (iii) *x*, *y* − 1, *z*]. Four of the cyanide ligands (C1/N1—C1/N1^i^, C2/N2—C2/N2^ii^, C3/N3—C4/N4 and C4/N4^iii^—C3/N3^iii^) are disordered over two sites with occupancies of 0.5. The Cu⋯Cu contact is shown as a dashed line.

Fig. 1 ▸ shows a fragment of the title compound, which is a polymeric copper complex with different coordination environments of the two crystallographically independent Cu^I^ ions. The Cu1 atom is coordinated to the N atom of a 2-eth­oxy­pyrazine mol­ecule [Cu1—N5 = 2.090 (4) Å]. Two other coordination positions are occupied by bridging cyanide groups, which are equally disordered over two sites, exchanging C and N atoms [Cu1—C1/N1 = 1.905 (4) Å and Cu1—C2/N2 = 1.888 (4) Å], thus forming an irregular triangular coordination geometry where the copper ion is displaced from the centre [C2/N2—Cu1—N5 = 108.9 (2)°, C1/N1—Cu1—N5 = 103.2 (2)° and C2/N2—Cu1—C1/N1 = 147.7 (2)°]. The Cu2 atom is coordinated by two cyanide ligands, which are also disordered over two sites with an occupancy of 0.5 for each C and N atom [Cu2—C3/N3 = 1.859 (5) Å and Cu2—C4/N4^iii^ = 1.841 (4) Å; symmetry codes: (i) −*x* + 1, *y*, −*z* + 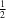; (ii) −*x* + 1, −*y*, −*z* + 1; (iii) *x*, *y* − 1, *z*] to form an almost linear chain [C4/N4^iii^—Cu2—C3/N3 = 170.5 (2)°]. The two Cu^I^ centres are connected through a Cu⋯Cu inter­action [Cu1—Cu2 = 2.7958 (13) Å] that could be inter­preted as a cuprophilic contact (Hermann *et al.*, 2001 ▸).

## Supra­molecular features   

**Figure 2:**
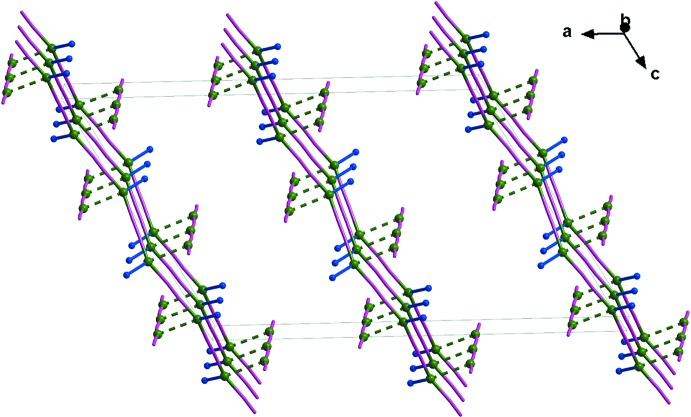
A view normal to the *ac* plane of the crystal structure of the title compound, showing the Cu⋯Cu contacts as dashed lines. 2-Eth­oxy­pyrazine rings (except for the N atoms connected to Cu1) and H atoms have been omitted for clarity. Colour code: Cu green, N blue and CN group magenta.

The crystal packing of the title compound (Fig. 2 ▸) consists of two types of orthogonal polymeric chains (the first involving the Cu1 atoms and parallel to the *c* axis and the second involving the Cu2 atoms and parallel to the *b* axis) inter­connected by Cu⋯Cu contacts and forming two-dimensional layers parallel to (100). The Cu⋯Cu contacts are almost perpendicular to the [Cu2(CN)] chains [C3/N3—Cu2—Cu1 = 89.8 (2)° and C4/N4^iii^—Cu2—Cu1 = 99.7 (2)°]. At the same time, the Cu2 atom occupies an axial position with respect to the triangular [N(CN)_2_] coordination environment of Cu1 [C1/N1—Cu1—Cu2 = 70.6 (2)° and C2/N2—Cu1—Cu2 = 87.6 (2)°]. The resulting metal–organic coordination framework is additionally stabilized by weak long-range (electrostatic-type) C—H⋯π inter­actions between cyanide groups and 2-eth­oxy­pyrazine rings (Aliev *et al.*, 2015 ▸; Table 1 ▸). Short Cu2⋯O1^iv^ contacts of 3.060 (3) Å are also observed [symmetry code: (iv) −*x* + 1, −*y* + 1, −*z* + 1].

## Database survey   

A search of the Cambridge Structural Database (CSD, Version 5.39, last update November 2017; Groom *et al.*, 2016 ▸) confirmed that the structure of the title complex has not been reported previously and revealed for the fragment –C≡N—Cu—C≡N– and an azine ligand attached to Cu (unsubstituted, substituted and fused azines) 128 structures, which are polymeric copper cyanide chains decorated with various co-ligands. Most of these co-ligands are derivatives of pyridine, piperidine, methyl­ene­tetra­mine and piperazine. In particular, the structure of *catena*-[penta­kis­(μ_2_-cyano)­tris­(1-phenyl­piperazine)penta­copper] (refcode VIYPOK; Pike *et al.*, 2014 ▸) contains five independent Cu atoms and five non­symmetrically disordered cyanides, and forms two independent one-dimensional chain sublattices, *i.e.* (CuCN)(PhPip) and (CuCN)_3_(PhPip), associated by Cu⋯Cu pairwise cuprophilic inter­actions, with distances of 2.5586 (10) and 2.6441 (10) Å. A search of the CSD for two C—N—Cu—C—N fragments with a defined Cu⋯Cu distance less than 2.8 Å gave 80 hits, among which is an example close to the title structure, *i.e.* *catena*-[(μ_2_-*N*-benzyl­piperazine-*N*,*N*′)tetra­kis­(μ_2_-cyano)­tetra­copper(I)] (refcode LOGWIO; Lim *et al.*, 2008 ▸), where the resulting network is composed of planar rows of undulating CuCN chains running roughly parallel to the *a* axis and crosslinked by bridging benzyl­piperazine ligands in the *c* direction, forming two-dimensional double sheets capped by nonbridging ligands. Two Cu⋯Cu inter­actions are present in the mentioned coordination polymer, with distances of 2.6650 (6) and 2.9644 (6) Å.

## Synthesis and crystallization   

Crystals of the title compound were obtained by slow diffusion within three layers in a 3 ml glass tube. The first layer was a solution of K[Cu(CN)_2_] (7.7 mg, 0.05 mmol) in 1 ml of H_2_O, the second layer was a H_2_O/EtOH mixture (1:1 *v*/*v*, 1 ml) and the third layer was a solution of 2-eth­oxy­pyrazine (3.1 mg, 0.025 mmol) in 0.5 ml of EtOH. After two weeks, colourless block-shaped crystals had formed in the middle layer. The crystals were kept under the mother solution prior to measurement.

## Refinement   

Crystal data, data collection and structure refinement details are summarized in Table 2 ▸. All H atoms were placed geometrically and refined as riding, with C—H = 0.93 Å and *U*_iso_(H) = 1.2*U*_eq_(C) for aromatic hydrogens, C—H = 0.97 Å and *U*_iso_(H) = 1.2*U*_eq_(C) for the CH_2_ group, and C—H = 0.96 Å and *U*_iso_(H) = 1.5*U*_eq_(C) for the CH_3_ group. A rotating model was used for the methyl group. All cyano ligands are disordered over two sites with occupancies of 0.5. The coordinates of C and N atoms sharing the same sites and their displacement ellipsoids were constrained to be the same.

## Supplementary Material

Crystal structure: contains datablock(s) I. DOI: 10.1107/S205698901901452X/rz5265sup1.cif

Structure factors: contains datablock(s) I. DOI: 10.1107/S205698901901452X/rz5265Isup2.hkl

CCDC references: 1961274, 1961274

Additional supporting information:  crystallographic information; 3D view; checkCIF report

## Figures and Tables

**Table 1:** Hydrogen-bond geometry (Å, °) *Cg* is the centroid of the C1/N1–C1^i^/N1^i^ cyano group [symmetry code: (i) −*x* + 1, *y*, −*z* + 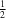]

*D*—H⋯*A*	*D*—H	H⋯*A*	*D*⋯*A*	*D*—H⋯*A*
C8—H8⋯*Cg*	0.93	2.93	3.558 (6)	126

**Table 2:** Experimental details

Crystal data	
Chemical formula	[Cu(CN)(C_6_H_8_N_2_O)][Cu(CN)]
*M*_r_	303.26
Crystal system, space group	Monoclinic, *C*2/*c*
Temperature (K)	293
*a*, *b*, *c* (Å)	26.840 (5), 4.830 (1), 18.620 (4)
β (°)	119.91 (3)
*V* (Å^3^)	2092.3 (9)
*Z*	8
Radiation type	Mo *K*α
μ (mm^−1^)	4.04
Crystal size (mm)	0.09 × 0.04 × 0.01

Data collection	
Diffractometer	Bruker SMART CCD
Absorption correction	Multi-scan (*SADABS*; Bruker, 2014 ▸)
*T*_min_, *T*_max_	0.630, 0.746
No. of measured, independent and observed [*I* > 2σ(*I*)] reflections	12437, 2498, 1396
*R*_int_	0.113
(sin θ/λ)_max_ (Å^−1^)	0.659

Refinement	
*R*[*F* ^2^ > 2σ(*F* ^2^)], *wR*(*F* ^2^), *S*	0.045, 0.091, 0.84
No. of reflections	2498
No. of parameters	137
H-atom treatment	H-atom parameters constrained
Δρ_max_, Δρ_min_ (e Å^−3^)	1.72, −0.73

Computer programs: *SAINT* (Bruker, 2013 ▸), *APEX2* (Bruker, 2013 ▸), *SHELXT* (Sheldrick, 2015*a* ▸), *SHELXL2017* (Sheldrick, 2015*b* ▸) and *OLEX2* (Dolomanov *et al.*, 2009 ▸).

## supplementary crystallographic information

### Crystal data

**Table:**

[Cu(CN)(C_6_H_8_N_2_O)][Cu(CN)]	*F*(000) = 1200
*M**_r_* = 303.26	*D*_x_ = 1.925 Mg m^−^^3^
Monoclinic, *C*2/*c*	Mo *K*α radiation, λ = 0.71073 Å
*a* = 26.840 (5) Å	Cell parameters from 1229 reflections
*b* = 4.830 (1) Å	θ = 3.1–22.8°
*c* = 18.620 (4) Å	µ = 4.04 mm^−^^1^
β = 119.91 (3)°	*T* = 293 K
*V* = 2092.3 (9) Å^3^	Block, colourless
*Z* = 8	0.09 × 0.04 × 0.01 mm

### Data collection

**Table:**

Bruker SMART CCD diffractometer	1396 reflections with *I* > 2σ(*I*)
ω scan	*R*_int_ = 0.113
Absorption correction: multi-scan (SADABS; Bruker, 2014)	θ_max_ = 27.9°, θ_min_ = 1.8°
*T*_min_ = 0.630, *T*_max_ = 0.746	*h* = −34→31
12437 measured reflections	*k* = −6→6
2498 independent reflections	*l* = −24→24

### Refinement

**Table:**

Refinement on *F*^2^	Primary atom site location: dual
Least-squares matrix: full	Hydrogen site location: inferred from neighbouring sites
*R*[*F*^2^ > 2σ(*F*^2^)] = 0.045	H-atom parameters constrained
*wR*(*F*^2^) = 0.091	*w* = 1/[σ^2^(*F*_o_^2^) + (0.0339*P*)^2^] where *P* = (*F*_o_^2^ + 2*F*_c_^2^)/3
*S* = 0.84	(Δ/σ)_max_ = 0.001
2498 reflections	Δρ_max_ = 1.72 e Å^−^^3^
137 parameters	Δρ_min_ = −0.73 e Å^−^^3^
0 restraints	

### Special details

**Table:**

Geometry. All esds (except the esd in the dihedral angle between two l.s. planes) are estimated using the full covariance matrix. The cell esds are taken into account individually in the estimation of esds in distances, angles and torsion angles; correlations between esds in cell parameters are only used when they are defined by crystal symmetry. An approximate (isotropic) treatment of cell esds is used for estimating esds involving l.s. planes.

### Fractional atomic coordinates and isotropic or equivalent isotropic displacement parameters (Å^2^)

**Table:**

	*x*	*y*	*z*	*U*_iso_*/*U*_eq_	Occ. (<1)
Cu1	0.48390 (3)	0.21483 (12)	0.37001 (4)	0.02665 (19)	
Cu2	0.60202 (3)	0.16869 (12)	0.43005 (4)	0.0350 (2)	
O1	0.31627 (15)	0.7706 (7)	0.3834 (2)	0.0307 (8)	
N5	0.42154 (19)	0.5236 (7)	0.3357 (2)	0.0242 (10)	
N6	0.3369 (2)	0.9360 (8)	0.2842 (3)	0.0303 (11)	
C4	0.6090 (2)	0.7890 (9)	0.4339 (3)	0.0284 (11)	0.5
C5	0.3898 (2)	0.5558 (10)	0.3714 (3)	0.0230 (12)	
H5	0.395884	0.440570	0.415076	0.028*	
C3	0.6081 (2)	0.5524 (10)	0.4335 (3)	0.0313 (12)	0.5
C8	0.4114 (2)	0.7025 (10)	0.2737 (3)	0.0256 (11)	
H8	0.432622	0.687906	0.246866	0.031*	
C6	0.3472 (2)	0.7606 (10)	0.3445 (3)	0.0269 (12)	
C7	0.3710 (3)	0.9009 (10)	0.2504 (3)	0.0320 (14)	
H7	0.366143	1.021425	0.208471	0.038*	
C9	0.2705 (2)	0.9697 (11)	0.3541 (4)	0.0355 (14)	
H9A	0.242718	0.935695	0.296265	0.043*	
H9B	0.285567	1.155698	0.359242	0.043*	
C10	0.2431 (3)	0.9374 (13)	0.4063 (4)	0.0437 (16)	
H10A	0.210384	1.058036	0.386082	0.066*	
H10B	0.270287	0.984284	0.462610	0.066*	
H10C	0.230857	0.749007	0.403666	0.066*	
N3	0.6081 (2)	0.5524 (10)	0.4335 (3)	0.0313 (12)	0.5
N4	0.6090 (2)	0.7890 (9)	0.4339 (3)	0.0284 (11)	0.5
C2	0.4969 (2)	0.0527 (9)	0.4702 (3)	0.0274 (12)	0.5
C1	0.4978 (2)	0.2054 (8)	0.2793 (3)	0.0254 (11)	0.5
N1	0.4978 (2)	0.2054 (8)	0.2793 (3)	0.0254 (11)	0.5
N2	0.4969 (2)	0.0527 (9)	0.4702 (3)	0.0274 (12)	0.5

### Atomic displacement parameters (Å^2^)

**Table:**

	*U*^11^	*U*^22^	*U*^33^	*U*^12^	*U*^13^	*U*^23^
Cu1	0.0346 (4)	0.0309 (4)	0.0197 (3)	0.0007 (3)	0.0175 (3)	0.0022 (3)
Cu2	0.0518 (5)	0.0196 (3)	0.0408 (5)	0.0005 (3)	0.0285 (4)	−0.0002 (3)
O1	0.030 (2)	0.041 (2)	0.0252 (19)	0.0082 (18)	0.0169 (17)	0.0077 (17)
N5	0.035 (3)	0.017 (2)	0.022 (2)	−0.0055 (19)	0.016 (2)	−0.0010 (18)
N6	0.035 (3)	0.022 (2)	0.031 (3)	−0.002 (2)	0.015 (3)	0.003 (2)
C4	0.035 (3)	0.026 (2)	0.024 (3)	0.000 (2)	0.015 (2)	−0.001 (2)
C5	0.033 (3)	0.022 (3)	0.017 (3)	0.000 (2)	0.015 (3)	0.002 (2)
C3	0.038 (3)	0.032 (3)	0.021 (3)	0.001 (3)	0.013 (3)	−0.003 (2)
C8	0.036 (3)	0.026 (3)	0.020 (3)	−0.004 (3)	0.018 (3)	0.000 (2)
C6	0.036 (3)	0.020 (2)	0.024 (3)	−0.006 (2)	0.015 (3)	−0.002 (2)
C7	0.043 (4)	0.026 (3)	0.030 (3)	−0.006 (3)	0.020 (3)	0.007 (2)
C9	0.030 (4)	0.043 (3)	0.031 (3)	0.007 (3)	0.013 (3)	0.004 (3)
C10	0.030 (4)	0.063 (4)	0.045 (4)	0.013 (3)	0.024 (3)	0.009 (3)
N3	0.038 (3)	0.032 (3)	0.021 (3)	0.001 (3)	0.013 (3)	−0.003 (2)
N4	0.035 (3)	0.026 (2)	0.024 (3)	0.000 (2)	0.015 (2)	−0.001 (2)
C2	0.033 (3)	0.029 (3)	0.021 (3)	0.000 (2)	0.014 (3)	0.000 (2)
C1	0.023 (3)	0.025 (2)	0.030 (3)	0.002 (2)	0.015 (2)	0.003 (2)
N1	0.023 (3)	0.025 (2)	0.030 (3)	0.002 (2)	0.015 (2)	0.003 (2)
N2	0.033 (3)	0.029 (3)	0.021 (3)	0.000 (2)	0.014 (3)	0.000 (2)

### Geometric parameters (Å, º)

**Table:**

Cu1—Cu2	2.7958 (13)	C5—H5	0.9300
Cu1—N5	2.090 (4)	C5—C6	1.402 (7)
Cu1—C2	1.888 (4)	C3—N4	1.143 (6)
Cu1—C1	1.905 (4)	C8—H8	0.9300
Cu1—N1	1.905 (4)	C8—C7	1.347 (7)
Cu1—N2	1.888 (4)	C7—H7	0.9300
Cu2—C3	1.859 (5)	C9—H9A	0.9700
Cu2—N3	1.859 (5)	C9—H9B	0.9700
O1—C6	1.347 (5)	C9—C10	1.492 (7)
O1—C9	1.436 (6)	C10—H10A	0.9600
N5—C5	1.325 (6)	C10—H10B	0.9600
N5—C8	1.357 (6)	C10—H10C	0.9600
N6—C6	1.322 (6)	C2—C2^i^	1.155 (8)
N6—C7	1.354 (6)	C1—C1^ii^	1.152 (8)
C4—N3	1.143 (6)		
			
N5—Cu1—Cu2	139.01 (11)	C7—C8—H8	119.5
C2—Cu1—Cu2	87.55 (16)	O1—C6—C5	116.4 (4)
C2—Cu1—N5	108.86 (18)	N6—C6—O1	120.7 (5)
C1—Cu1—Cu2	70.59 (14)	N6—C6—C5	122.9 (5)
C1—Cu1—N5	103.17 (17)	N6—C7—H7	117.9
N1—Cu1—Cu2	70.59 (14)	C8—C7—N6	124.2 (5)
N1—Cu1—N5	103.17 (17)	C8—C7—H7	117.9
N2—Cu1—Cu2	87.55 (16)	O1—C9—H9A	110.4
N2—Cu1—N5	108.86 (18)	O1—C9—H9B	110.4
C3—Cu2—Cu1	89.85 (17)	O1—C9—C10	106.7 (4)
N3—Cu2—Cu1	89.85 (17)	H9A—C9—H9B	108.6
C6—O1—C9	117.2 (4)	C10—C9—H9A	110.4
C5—N5—Cu1	123.2 (3)	C10—C9—H9B	110.4
C5—N5—C8	116.4 (4)	C9—C10—H10A	109.5
C8—N5—Cu1	120.3 (3)	C9—C10—H10B	109.5
C6—N6—C7	114.3 (4)	C9—C10—H10C	109.5
N5—C5—H5	119.4	H10A—C10—H10B	109.5
N5—C5—C6	121.2 (4)	H10A—C10—H10C	109.5
C6—C5—H5	119.4	H10B—C10—H10C	109.5
N4—C3—Cu2	176.6 (5)	C4—N3—Cu2	176.6 (5)
N5—C8—H8	119.5	C2^i^—C2—Cu1	177.4 (7)
C7—C8—N5	121.0 (5)	C1^ii^—C1—Cu1	175.0 (6)

Symmetry codes: (i) −*x*+1, −*y*, −*z*+1; (ii) −*x*+1, *y*, −*z*+1/2.

### Hydrogen-bond geometry (Å, º)

Cg is the centroid of the C1/N1–C1^i^/N1^i^ cyano group (symmetry code: (i) 1-x, y, 1/2-z)

**Table:**

*D*—H···*A*	*D*—H	H···*A*	*D*···*A*	*D*—H···*A*
C8—H8···*Cg*	0.93	2.93	3.558 (6)	126
